# The Impact of Acute or Chronic Alcohol Intake on the NF-κB Signaling Pathway in Alcohol-Related Liver Disease

**DOI:** 10.3390/ijms21249407

**Published:** 2020-12-10

**Authors:** Aleksander J. Nowak, Borna Relja

**Affiliations:** 1 Experimental Radiology, University Clinic for Radiology and Nuclear Medicine, Leipziger Strasse 44, 39120 Magdeburg, Germany; aleksander.nowak@med.ovgu.de; 2 Medical Faculty, Otto-von-Guericke-University Magdeburg, Leipziger Strasse 44, 39120 Magdeburg, Germany

**Keywords:** chronic alcohol intake, acute alcohol intake, immunology, alcoholic liver disease, signaling, nuclear factor kappa B, A20, OTUB-1

## Abstract

Ethanol misuse is frequently associated with a multitude of profound medical conditions, contributing to health-, individual- and social-related damage. A particularly dangerous threat from this classification is coined as alcoholic liver disease (ALD), a liver condition caused by prolonged alcohol overconsumption, involving several pathological stages induced by alcohol metabolic byproducts and sustained cellular intoxication. Molecular, pathological mechanisms of ALD principally root in the innate immunity system and are especially associated with enhanced functionality of the nuclear factor kappa-light-chain-enhancer of activated B cells (NF-κB) pathway. NF-κB is an interesting and convoluted DNA transcription regulator, promoting both anti-inflammatory and pro-inflammatory gene expression. Thus, the abundancy of studies in recent years underlines the importance of NF-κB in inflammatory responses and the mechanistic stimulation of inner molecular motifs within the factor components. Hereby, in the following review, we would like to put emphasis on the correlation between the NF-κB inflammation signaling pathway and ALD progression. We will provide the reader with the current knowledge regarding the chronic and acute alcohol consumption patterns, the molecular mechanisms of ALD development, the involvement of the NF-κB pathway and its enzymatic regulators. Therefore, we review various experimental in vitro and in vivo studies regarding the research on ALD, including the recent active compound treatments and the genetic modification approach. Furthermore, our investigation covers a few human studies.

## 1. Introduction

Ethanol (ethylic alcohol, EtOH) in the form of various and diversified beverages is one of the world’s most commonly consumed active ingredients in drinks, alongside caffeine [1]. Besides recreational and culturally related purposes, ethanol is also a ubiquitous chemical compound applied frequently as a disinfectant, an antiseptic, a solvent and an antidotal drug, as well as being used as a fuel base in the industry [2]. In the short term, alcohol exhibits multiple biological effects on the human organism, including a severe psychoactive stimulation (by disrupting signaling pathways in the brain, leading to euphoria, disinhibition or moods swings) [3], arrhythmia, ataxia or analgesia [4]. However, chronic EtOH consumption can lead to several complex diseases like alcoholic gastritis, alcoholic myopathy, alcoholic cardiomyopathy, alcoholic neuropathy, alcoholic pancreatitis and alcoholic liver disease (ALD) [5,6]. The latter one is a complex and progressive condition that comprises many stages regarding the advancement of the disease, where the treatment possibilities range from a simple alcoholic abstain in its milder forms to a liver transplantation in the acute and terminal phases [7]. The multi-layered pathogenesis of ALD is still not comprehensively understood, especially the direct alterations of signaling pathways in the liver cells, which stimulate an immune response and abundant release of pro-inflammatory factors, including tumor necrosis alpha (TNFα) and interleukin 1-beta (IL-1β) [8]. This leads to the change in expression levels of nuclear factor kappa-light-chain-enhancer of activated B cells (NF-κB), a crucial transcription factor complex [9]. NF-κB is an important signaling cascade regulator that controls the genes responsible for innate and adaptive immunological responses [10]. It is believed that chronic alcoholic disease induces heavy shifts in metabolism and signaling of both parenchymal and non-parenchymal liver cells, thus leading to accumulation of fatty acids in the liver and causing the disease progression [11]. This review focuses predominantly on a correlation between alcoholic liver disease and any alternations in function of the NF-κB regulatory complex during induced liver alcoholic injury in animal models. It is assumed that inner mechanisms of the ALD pathogenesis lie profoundly within the various liver cells via NF-κB signaling malfunctions [12]. Therefore, we surveyed the most recent literature papers regarding the topic, contributing to the establishment of a novel discussion environment for possible treatments and research directions in the future of alcoholic liver disease investigations [13].

## 2. Review Criteria

The data presented and analyzed in this review article were acquired through perusing various scientific databases (primarily PubMed), by exerting the Web of Science search tool and Google Scholar search engine, together with applying proper Boolean operators and by recombining the search terms to narrow the results. The assortment of used keywords and terms includes the following: “alcohol*”, “liver”, “disease*”, “alcohol-related”, “ALD”, “ethanol OR EtOH”, “(nuclear NEAR/1 factor NEAR/1 kappa NEAR/1 b OR NF-κB)”, “(chronic OR acute) AND intake”, “acute-on-chronic”, “liver NEAR/1 inflammation”, “cirrhosis”, “fibrosis”, “hepatic NEAR/1 steatosis”, “HCC or hepatocellular NEAR/1 carcinoma”, “alcoholic NEAR/1 hepatitis”, “A20”, “OTUB1”, “DUB* OR deubiquitin* AND enzyme*”. Articles without certain relevance value for the review subject were excluded from the paper after the consideration study. There was no timeframe limit for the publication date, although the articles published in the last 5 years had preference. Only completely English-written texts are presented here. Furthermore, we surveyed through reference lists of the articles to search for additional relevant data.

## 3. Alcohol Intake and Its Influence on the Liver

Alcohol consumption in miscellaneous forms is constantly present in almost every part of humankind history, dating back as far as 11,000 years BC [14]. Nowadays, drinking alcoholic beverages in assorted ways is an integral part of cultures across the world and alcohol is perceived as a recreational soft drug by modern society [15]. In addition, the whole global alcohol industry market share is accounted for over USD 1 trillion [16]. According to the reports of the USA Substance Abuse and Mental Health Services Administration in 2018 from the National Survey on Drug Use and Health, it is estimated that over 80% of adults aged 18 or more consumed alcohol at least once in their lives, while over 25% of the surveyed group admitted to binge drinking [17]. The actual scale of worldwide alcohol usage is difficult to assume entirely by flat numbers, however, the recent studies assigned about 2.3 billion people worldwide to be active drinkers [18]. There are various different alcohol drinking patterns, ranging from occasional mild consumption to addiction-driven heavy alcohol abuse, however, the two most relevant ones for studying alcohol influence are binge (acute) and prolonged (chronic) drinking habits [19]. Binge drinking is defined as intense alcohol consumption in a short period of time (2 h or below) that concludes in a blood alcohol concentration (BAC) level equal to or above 0.08% [20]. Chronic alcohol abuse is mostly understood as prolonged drinking, often associated with psychical dependency on alcohol or even constituting already developed alcoholism [21]. However, the exact definitions of drinking patterns are blurred and unclear with a magnitude of intermediate settings, e.g., extreme binge, chronic binge or heavy drinking [22]. In animal models, binge setting is usually correlated with an alcohol dosage resulting in a final intake of 5–8 g of EtOH per kg of the subject’s body weight in a one-off or repeated manner [22]. Meanwhile, chronic alcohol research in animal subjects constitutes various models, including the Lieber–DeCarli diet or the Tsukamoto–French model [23]. Nevertheless, this review will not cover the current rodent EtOH research models in use (for a detailed description, please refer to [23]). Importantly, the different effects of short-term and prolonged alcohol intake by humans are still under investigation in different settings [24], although it is thus far not disputable that alcohol leads to damage on several layers [25]. In consonance with Nutt et al., it is presumed that damage caused to society by alcohol drinking is more severe than the damage inflicted by other health-impactful drugs, such as cocaine or opioids and their derivatives [26]. Thus, alcohol drinking leads to high morbidity and mortality, both in the long and short term, and constitutes a grave global problem [27], while ALD with its detrimental disease development, as described later in the text, is one of foremost reasons of mortality [28]. However, first and foremost, for the sake of reading coherency, it is important to define ALD as a medical condition.

### 3.1. Alcohol-Related Liver Conditions

The straightforward description of ALD as a medical condition is not so apparent and obvious, considering the wide spectrum of its classified disorders, including simple hepatic steatosis, alcoholic steatohepatitis, hepatic fibrosis or hepatic cirrhosis [29]. The first stage of chronic alcohol disease is hepatic steatosis. It is characterized by the accumulation of fat molecules in the liver tissue due to excessive effects of toxic EtOH metabolites and enhanced lipogenesis, which means an overproduction of fatty acids [30]. Hepatic steatosis is a reversible stage, and the liver is able to regenerate itself upon alcohol withdrawal [31]. On the other hand, untreated steatosis can lead to alcoholic hepatitis, a condition of continuous liver inflammation with the frequent appearances of Mallory bodies in the liver tissue [32]. Caused by chronic inflammation, prolonged, neglected hepatitis can lead to hepatic fibrosis, where collagen tissue replaces the healthy cells in the liver, resulting in the formation of a scar tissue that is characterized by a dysfunctional cellular formation [33]. Hepatic cirrhosis is usually the last stage of the disease progression that is caused by the continuous tissue scarification and results in a liver function failure—at this point, the only viable treatment option is a liver transplantation, supported by alcohol withdrawal and steroids therapy [34]. Additionally, chronic drinking can lead to hepatocellular carcinoma (HCC), often developing simultaneously with advanced cirrhosis [35]. The currently known and described mechanisms of the disease progression will be addressed later in this review. As the name of the condition suggests, the disease manifests its progression primarily in the liver tissue and it is the major axis of all the pathological changes in ALD.

### 3.2. The Liver—Structure, Components and Function

The liver is the essential organ in the biology of ALD development and progression, fulfilling immunological, metabolic, productive, secretive, digestive, endocrinal and other impactful roles in vertebrates [36]. The liver tissue structure is complex, as its numerous functions require multiple cellular players for efficiency. The commonly binucleated [37] parenchymal cell type of the liver and the most abundant cellular fraction (about 60% of the whole organ mass [38]) consists of hepatocytes, a group of sizable epithelial cells with multiple mitochondria present. Hepatocytes synthesize essential proteins, bile acid and cholesterol, modify carbohydrates and are responsible for detoxification processes, including EtOH metabolism [39]. Another liver fraction is composed of so-called Ito cells, likewise recognizable as hepatic stellate cells (HSCs), residing in the space of Disse [40]. Their physiological role has not yet been completely elucidated—however, HSCs are capable of storing vitamin A derivatives and participate in the pathological collagen secretion during hepatic fibrosis, thereby promoting the formation of the scar tissue. Consecutively, untreated fibrosis leads to a more dreadful condition of hepatic cirrhosis that is characterized by the permanent displacement of normal liver tissue with mutilated, scarred collagen matter [41,42]. The macrophages of the liver, located in the vascular spaces of sinusoids [43], are widely acknowledged as Kupffer cells and their major responsibility is the removal of pathogens and phagocytosis of dead erythrocytes. Moreover, Kupffer cells are activated during alcohol-induced injury and play a pivotal role in the progression of liver inflammation in chronic alcoholic disease [44]. The last but not least are liver sinusoidal endothelial cells (LSEC) that line the surfaces of hepatic blood vessels. LSECs are equipped with fenestrae that allow them to clear sinusoidal waste [45]. Since the liver processes the majority of alcohol volumes acquired by the body, over 80% of the whole intake [46], all of the aforementioned cell types are important components of pathological ALD progression. However, the enzymatic core of EtOH metabolism resides in hepatocytes, and EtOH metabolic intermediates are the key players in ALD development.

### 3.3. Toxicity of Alcohol to the Liver

Breakdown of EtOH to its metabolites occurs through three oxidative catabolic pathways. In parenchymal liver cells, EtOH is mainly oxidized by the cytosol-based enzyme alcohol dehydrogenase (ADH), which converts the hydroxyl-based EtOH compound into its metabolic intermediate ethanal (MeCHO, acetaldehyde), which is characterized by the presence of the functional formyl group [46]. Another oxidation pathway consists of the microsomal ethanol oxidation system (MEOS), which requires the cytochrome P450 2E1 (CYP2E1) for its functionality. The whole system is located in the endoplasmic reticulum (ER) and it is especially active during prolonged, chronic alcohol intake and operates as a supporting mechanism for the main ADH-based oxidation [47]. The extrahepatic cells not containing ADH (e.g., brain cells) are capable of metabolizing EtOH to MeCHO in a tertiary pathway by exhibiting the catalase conversion activity, which is ordinarily responsible for decomposition of hydrogen peroxide (H_2_O_2_) [48]. Regardless of the manner of the initial oxidation, acetaldehyde is afterwards reduced to acetic acid (MeCOOH) by the aldehyde dehydrogenase (ALDH) and thus excreted from the organism [49]. Interestingly, ALDH occurs in two catalytically equivalent isoforms (isozymes) located in the cytosol and mitochondria [50]. East Asian populations do not possess the mitochondrial isozyme, and thus the syndromes of acute alcohol intoxication are noticeably more onerous for Asians than for, e.g., Caucasians. In addition, mitochondrial homolog deficiency increases susceptibility to HCC [50,51].

MeCHO is an exceedingly toxic and noxious compound, as well as a very potent carcinogen [52]. MeCHO damages DNA by causing development of DNA adducts, leading to substitutions, cross-linking or double-strand breaks (DSBs) [53]. In higher concentrations, MeCHO also disrupts the pulmonary structures and harms the respiratory tract [54]. However, the toxicity of EtOH consumption is not only limited to the sole in-cell accumulation of MeCHO. For instance, the electrochemical redox reaction of EtOH/MeCHO metabolism requires an oxidizing agent in the form of nicotinamide adenine dinucleotide (NAD^+^) to function properly. After accepting the reducing equivalents from EtOH/MeCHO molecules during the reaction, the NAD^+^ molecule reduces itself to NADH [55]. This reaction severely depletes the available population of NAD^+^ and disrupts the essential NAD^+^/NADH inner balance ratio. Hence, the disruption causes the inhibition of such processes as ketogenesis, glycolysis and fatty acid oxidation, thus favoring the aforementioned lipogenesis and gluconeogenesis and therefore leading to steatosis development [46]. Furthermore, MeCHO levels negatively affect the availability of glutathione molecules (GSH) that are important antioxidant agents [56]. GSH serves as the natural protectant from reactive oxygen species (ROS) [57]. Consequently, another dangerous aspect of alcohol intake is the accumulation of the aforementioned ROS, such as H_2_O_2_, in hepatocytes and Kupffer cells. The abundancy of ROS causes oxidative stress and ER stress, as well as fatty acids overproduction and accumulation, leading to hepatic steatosis progression [58]. The adipose-derived protein hormone known as adiponectin regulates fatty acids metabolism in the liver by suppressing hepatic lipogenesis and enhancing β-oxidation [59]. During chronic alcohol exposure, the adiponectin protein level is downregulated, limiting fatty acids oxidation and changing the lipids balance even further, hence amplifying lipogenesis [60]. Moreover, the pathogenesis of ALD does not relate solely to the pure chemical properties of EtOH and its metabolites, since they directly affect diverse cellular components. As the liver is an immune organ itself, EtOH consumption is associated with the activation of both several signaling pathways and the innate immune system, thus initiating a multi-cascade of different biochemical actions and immunological responses [61]. One important process in the initiation of liver inflammation is the ability of EtOH to downregulate the expression of tight junction proteins, e.g., occludin protein, thereby loosening tight junctions between intestinal epithelial barrier cells in the gut [62,63]. This permits the transposition of microbe-derived constituents (most importantly, endotoxin—lipopolysaccharide, LPS) via the portal vein to the liver [64]. Moreover, EtOH also causes a dysbacteriosis of the gut bacterial flora, suppressing beneficial and symbiotic species in favor of less benignant bacterial phyla that produce higher quantities of dangerous endotoxins [65]. All these factors together stimulate the liver macrophages (Kupffer cells) to produce and release pro-inflammatory cytokines and chemokines, leading to the recruitment of other immune cells and triggering an inflammatory signal cascade throughout the liver tissue [66]. Additionally, there are several other pathological conditions of alcohol misusage, e.g., the initial loss of liver endothelial cells fenestrae in ALD [67] that increases during chronic fibrosis and concludes in the strengthening of the LSECs defenestration effect [68]. This phenomenon leads to the enhancement of the inflammatory status or hepatic encephalopathy, an ill-understood alcoholic liver-related brain malfunction [69]. Yet it is important to state that drinking behavior significantly affects the briefly described changes in the liver: binge drinking may cause enhanced inflammation in situ and alternations that are acute, although rather reversible [70], while chronic consumption leads to constant liver inflammation, permanent changes and advanced pathological conditions of the organ [71]. Nevertheless, the cause diversity of alcohol-related pathological conditions of the liver roots deeply in the NF-κB molecular signaling pathway and its functionality.

## 4. The Role of NF-κB in the Innate Immune Response

Innate immunity is the first line of immunological defense against pathogen-derived or endogenous danger signals, often acting quickly to eliminate them [72]. To sensor these triggers of inflammation, the innate immune system is equipped with pattern recognition receptors (PRRs), which are expressed on the surface of various cell types, i.e., macrophages, neutrophils, epithelial cells or dendritic cells [73]. These receptors, that were preserved over the course of evolution, are sensitive to so-called pathogen-associated molecular patterns (PAMPs) that are derived from bacterial or viral pathogens, i.e., the aforementioned LPS, the flagellin protein [74], peptidoglycans [75] or viral double-stranded RNA [76]. PRRs are also responsive to the molecular components released by malfunctioning and injured cells upon cellular stress, including S100 proteins, heat shock proteins, histones or F-actin [77]. According to the assumed nomenclature, these factors are coined as damaged-associated molecular patterns (DAMPs) (for further insights on DAMPs, please refer to our other review [78]). The recognition of either PAMPs or DAMPs by the PRRs induces signaling cascades, which leads to the release of signal transductors or enhancers and strengthens the immunological response to the particularly patterned ligand. One of most distinguishable, exemplary and notorious PRRs is a family of toll-like receptors (TLRs), which when activated by the respective adaptor molecules induce a signal transduction via transcription factors [79]. One of the most important and prominent transcription factors involved in the regulation of inflammation is NF-κB. This protein complex belongs to a group of transcription factors and it regulates the expression of many varied genes—most prominently, pro-inflammatory genes, inducing release of various chemokines and cytokines, thus resulting in inflammation [80]. Furthermore, NF-κB is involved in the co-regulation of pro-proliferative proteins, e.g., cyclin D and the G1 phase checkpoint regulator, thus controlling the cell growth cycle [81]. It also plays an important role in the mediation of apoptosis through a TNFα-mediated pathway [82] and there is also evidence of its participation in neurogenesis [83]. In addition, it is believed to be the regulator of c-myc levels, a known oncogenic protein [84], which further supports NF-κB’s oncogenic involvement in tumorigenesis [85]. This versatile transcription system also participates in the crosstalk with other relevant pathways, e.g., JNK or p53 pathways [86]. This is possibly due to the binding specificity of subunits belonging to the NF-κB family complex.

The NF-κB family constitutes five structurally conservative members: NF-κB1 (p50), NF-κB2 (p52), RelA (p65), RelB and c-Rel [87]. These components mediate the transcription by mutually binding and thus creating specific homo- and heterodimers, which concludes in transactivation or transrepression of target genes [88]. NF-κB resides in the cytosol, inactivated by the repression of the molecular inhibitors of the nuclear factor kappa B (IκB) family, which contain ankyrin repeats in their structural domains that have a strong affinity towards the Rel homology domain (RHD) in NF-κB proteins [89]. Proteins p50 and p52 derive from precursor proteins p105 and p100, respectively, and those precursors exhibit an inhibitory function similar to IκB-proteins [90]. There are two main pathways of NF-κB activation, and both of these lead to dissociation of IκB from the complex and the assembly of hetero- or homodimers that transfer to the nucleus and bind to the respective DNA target [91]. Canonical pathway signaling occurs due to a signal transduced from either TLR/myeloid differentiation primary response 88 protein (TLR/MyD88), interleukin-1 receptor associated kinase (IL1R/IRAK) or tumor necrosis factor receptor 2/terminal TNFR-associated factor 2 (TNFR/TRAF2) surface receptor complexes, T cell receptors (TCRs), B cell receptors (BCRs) and growth factor receptor (GFRs) [92]. Via binding of the respective ligands, the signal is transferred downstream and activates the inhibitor of nuclear factor kappa B kinases (IKK) [93]. The members of IKKs include catalytic units IKKα and IKKβ. Furthermore, there is an important regulatory unit or NF-κB essential modulator (NEMO or IKKγ) and the canonical NF-κB pathway functionality depends on the NEMO protein [94]. Consecutively, the kinase catalytic subunits catalyze the phosphorylation and poly-ubiquitination of IκB proteins, causing a dissociation from the complex. Therefore, this allows NF-κB dimers (usually the p50:RelA heterodimer) to relocate to the nucleus and initiate genes transcription [95]. A non-canonical pathway covers a similar mechanism, although it is NEMO- and IKKβ-independent and it does not require the aforementioned proteins’ involvement to activate the NF-κB dimers [96]. The signal starts from the binding of respective ligands to the cluster of differentiation 40 protein (CD40) on the surface of antigen-presenting cells or B cell activating factor receptor (BAFFR) or lymphotoxin beta-receptor (LTBR) [97]. Instead of activating the IKKαβγ complex, the signal activates the NF-κB-inducing kinase (NIK) which leads to phosphorylation of the IKKα homodimer. The activated homodimer catalyzes the processing of the p100 precursor into the p52 NF-κB protein and the whole process concludes in the formation of the p52:RelB heterodimer. In consequence, the nucleus translocation analogically mimics the canonical pathway with the operative heterodimer regulating downstream genes in the nucleus [98]. The importance of NF-κB activation lies in its position as a central regulator of the many cross-linking signaling pathways, its role as an ultimate responder of the organism to environmental changes, cellular stress and pathogen manifestations and its ability to control the cells’ proliferation or apoptosis proceedings. The main (canonical) pathway activated by various inflammatory signals (i.e., pro-inflammatory cytokines) leads to expression of the following cytokines: interleukins 4, 5 and 6 (IL-4, IL-5, IL-6), as well as IL-1β and TNFα; and chemokines: macrophage inflammatory protein (MIP-1), interleukin 8 (IL-8) or chemokine ligand 5 (RANTES), mainly involved in various chronic diseases [99]. For detailed insight details on NF-κB’s and cytokines’ involvement in chronic diseases, we recommend referring to [99].

The activation of the NF-κB signaling pathway also includes the expression of the following enzymatic proteins: nitric oxide synthase (iNOS) and cyclooxygenase 2 (COX-2); adhesion molecules: vascular cell adhesion protein 1 and 2 (VCAM-1, VCAM-2); and, finally, other pro-inflammatory compounds like C-reactive protein [100,101,102,103]. All of these factors result in multi-coordination and the enhancement of the inflammatory innate response [104]. Following the previous sentence, the induced NF-κB canonical pathway enhances the expression of the aforementioned cytokines and chemokines in many immune cells, evoking important molecular effects [10]. For example, the main effectors of NF-κB induction are neutrophils, which are the first granulocytes to react at the site of the on-going inflammation process following chemical signals—in the liver, they are mainly responsible for the damage to parenchymal cells [105]. The contact between neutrophils and hepatocytes through intercellular adhesion molecules (ICAM-1) present on the surface of the latter concludes in necrotic death of hepatocytes through the respiratory burst of ROS [105]. Moreover, NF-κB leads to the activation of dendritic cells, which present PAMP- and DAMP-derived antigens to other immune cells and contribute to ever-increasing, at this stage, production of pro-inflammatory transmitters [106]. On the other hand, the NF-κB-driven through PRR activation macrophages polarize into an M1 state, which results in even more increased overexpression of pro-inflammatory cytokines (primarily IL-6 and TNFα) [107]. In addition, macrophages help neutrophils in eliminating pathogens, dead or dying cells and cellular debris that were beforehand marked for phagocytosis due to on-going inflammation, e.g., by the aforementioned C-reactive protein [108]. During alcohol-related inflammation in the liver, the NF-κB-driven polarization to an M1 state of residing Kupffer cells leads to neutrophilic infiltration in the liver [109]. The induced expression of VCAM proteins in endothelial cells by cytokines stimulation allows lymphocytes (e.g., natural killer cells) adhesion to the endothelium and kills the affected cell [110], while iNOS produces abundantly pro-inflammatory nitric oxide, contributing to oxidative stress and cells toxicity by attracting neutrophils [111]. COX-2 is responsible for production of prostaglandins from arachidonic acid, which act as attractants for T cells [112], while the culmination of overexpression of cytokines stimulates T cells to differentiate into various functions, i.e., helper, killer or memory cells [113]. T cells and B cells are the part of adaptive immune system, not covered by this review (for the insights on the correlation between alcohol and the adaptive immune system, see [114]). The pro-inflammatory signals can also be transduced through the non-canonical pathway of NF-κB activation, which supports the immune system functions alongside the main canonical activation way [115]. The non-canonical pathway, triggered by receptors of the aforementioned ligands, contributes to the response by the release of differentiated proteins, e.g., B cell activating factor (BAFF), B lymphocyte chemoattractant (BLC), secondary lymphoid tissue chemokine (SLC), stromal cell-derived factor 1 (SDF-1) and EBI1 ligand chemokine (ELC) [10]. Intriguingly, all of these factors regulate the lymphoid secondary organ development, together with the maturation of immune cells and the differentiation of macrophages [10,116]. Moreover, the non-canonical pathway supports dendritic cells maturation to antigen-presenting cells through elevated levels of the RelB subunit protein [117].

**Figure 1:**
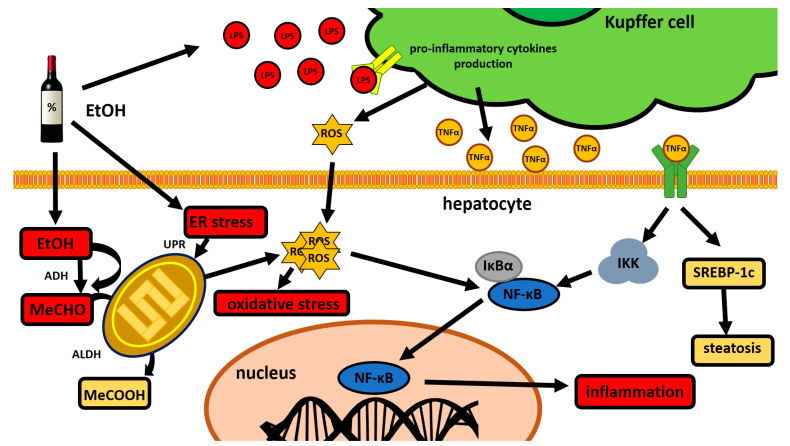
A schematic representation of the alcohol-related mechanism of liver injury during prolonged EtOH consumption. MeCHO causes an excessive release of reactive oxygen species (ROS) which leads to cellular damage and induction of the NF-κB pro-inflammatory pathway. Additionally, alcohol consumption results in gut permeability, which concludes in Kupffer cells stimulation by, i.e., endotoxins (LPS), which stimulates NF-κB-induced inflammation even further and also favors fatty acids overproduction. ADH—alcohol dehydrogenase, ALDH—aldehyde dehydrogenase, ER—endoplasmic reticulum, EtOH—ethanol, IKK—inhibitor of nuclear factor kappa B kinase complex, IκBα—inhibitor of nuclear factor kappa B alpha, LPS—lipopolysaccharide, MeCHO—acetaldehyde, MeCOOH—acetic acid, NF-κB—nuclear factor kappa-light-chain-enhancer of activated B cells, ROS—reactive oxygen species, SREBP-1c—sterol regulatory element-binding protein (for further insight on SREBP-1c, please refer to Section 6.1), TNFR—TNF receptor, TNFα—tumor necrosis factor alpha, UPR—unfolded protein response.

In summary, the ubiquitous transcription factor NF-κB is capable of inducing expression of a whole repertoire of various pro-inflammatory genes in both parenchymal and immune cells. The transmitter-induced signaling pathways and activation of immune cells conclude finally in the neutrophil infiltration and progressive death of hepatocytes, contributing in the end to the backbone of the liver injury mechanism of the liver. For the brief graphical representation of EtOH impact of NF-κB in the liver cells, please see Figure 1. 

Additionally, for the curious readership, we provide another review covering the correlation between innate immunity and the multi-layered alcohol impact more thoroughly [118].

## 5. NF-κB-Regulating Molecular Factors

The group of deubiquitinating enzymes (DUBs) is a paramount and vast class of protein superfamilies that altogether act as varied biochemical controllers by catalytically cleaving ubiquitin (Ub), a ubiquitous regulatory protein, from its molecular substrate targets [119]. The DUBs group consists of cysteine proteases and metalloproteases [120] that mechanistically process ubiquitin precursors to ubiquitin and detach the mono-ubiquitin or poly-ubiquitin chains from the corresponding proteins. All of these biochemical reactions are classified in the repertoire of post-translation modifications (PTMs) that frequently occur following protein biosynthesis in ribosomes [121]. A particularly fascinating DUB in regard to the NF-κB- and ALD-related discussion is a protein known as A20, discovered by Dixit et al. in the early 1990s [122]. A20, also known as tumor necrosis factor alpha-induced protein 3 (TNFAIP3 [123]), is expressed upon oxidative stress or inflammatory stimulation with, e.g., LPS, by a dozen of differentiated cell types, e.g., pancreatic cells [124], hepatocytes [125] or endothelial cells [122]. There is evidence that its influence on NF-κB is predominately negative, as it does contribute to the negative regulatory feedback loop that aims to refurbish the inflammatory condition to its unedited state, thus A20 functions as the NF-κB activity inhibitor [123]. Both of the A20 ubiquitin activities (E3 ubiquitination and DUB activity—the deubiquitinating activity from the amino-terminal ovarian tumor (OTU) domain) seem to have an enormous influence on NF-κB inhibition, although the exact explanation to this phenomenon still requires further investigation [126]. It was previously described that A20 removes lysine63-linked ubiquitin chains from an important TNF pathway receptor-interacting Ser-Thr protein kinase 1 (RIPK1) or NEMO from the IKK units and is capable of attaching lysine48-linked ubiquitin chains on RIPK1 or TNF-receptor, thus inducing the proteasome degradation process of marked targets [127]. However, there is experimental evidence published suggesting that the deubiquitinating activity of A20 might be marginal for NF-κB downregulation, as it presumably does not negatively affect inflammation progression in mice with inactivated DUB and E3 domains [128]. However, A20-deficient mice exhibit severe systemic inflammation and a high perinatal mortality rate [129]. To target the issue, Martens et al. generated recombined mice mutants, deficient, simultaneously, in the zinc finger 7 (ZnF7, poly-ubiquitin binding activity) and zinc finger 4 (ZnF4, the aforementioned E3 ligase activity) domains of A20 [130]. The study concluded in the acquirement of mice mutants exhibiting the same lethality levels and acute inflammation symptoms as the full-length A20-deficient mice [130]. Interestingly, the experimental group of Razani et al. achieved similar conclusions and results [131]. This underlies the pivotal role of A20 zinc finger domains and the wholesome ubiquitin-binding properties in inflammation protection. However, much has yet to be investigated, as it is unclear how A20 drives NF-κB regulation and inflammasome activation, especially in the setting of alcohol-induced liver injury. In terms of focused liver cell-based studies with a full-length A20 gene knockout (KO) approach, it was proven that A20 deficiency in HSCs causes lymphopenia, increased postnatal lethality and anemia in mice, which is a consequence of the loss of HSCs quiescence [132]. The A20-KO in hepatocytes results in chronic liver inflammation, as well as increased inflammation induction by pro-inflammatory stimulants like LPS or TNF, as well as enhanced susceptibility to HCC [125]. The protective properties of A20 towards HCC progression have been shown in another study, where A20 acts as an E3 Ub-ligase for a liver-type phosphofructokinase (PFKL) [133]. This leads to the inhibition of glycolysis and ceases cancerous proliferation, hence the evidence for the potential antitumor role of A20 emerges [133]. A20 was also suspected earlier to suppress HCC by its Twist1 inhibition activity [134]. To our knowledge, there are no investigations solely oriented to the role of A20 in the pathogenesis of ALD yet. Moreover, there is another important player in NF-κB inducible signaling also belonging to the DUBs, the so-called ubiquitin aldehyde binding 1 protein (OTUB-1, a member of the otubains family, the group of cysteine proteases [135]). It was reported already that OTUB-1 is an E2 ligase ubiquitin conjugated enzyme 13 (UBC13) [136] regulator, where UBC13 acts as a regulatory factor in the ring finger protein 168 (RFP168) signaling pathway [137], which participates in double-strand DNA breaks repair [138]. In consequence, OTUB-1 exhibits inhibitory properties on UBC13 [139]. Recently, it was also observed that OTUB-1 promotes NF-κB activation in dendritic cells during stimulated inflammatory conditions by lysine48 deubiquitination of UBC13 [140]. Additionally, there is evidence that OTUB-1 deficiency leads to a gradual degradation of the NF-κB precursor protein p100 to p52, which culminates in NF-κB activity induction [141]. These studies underline the OTUB-1 function as an important DUB-based NF-κB regulator, although a comprehensive experimental study in that regard should still be conducted.

**Figure 2:**
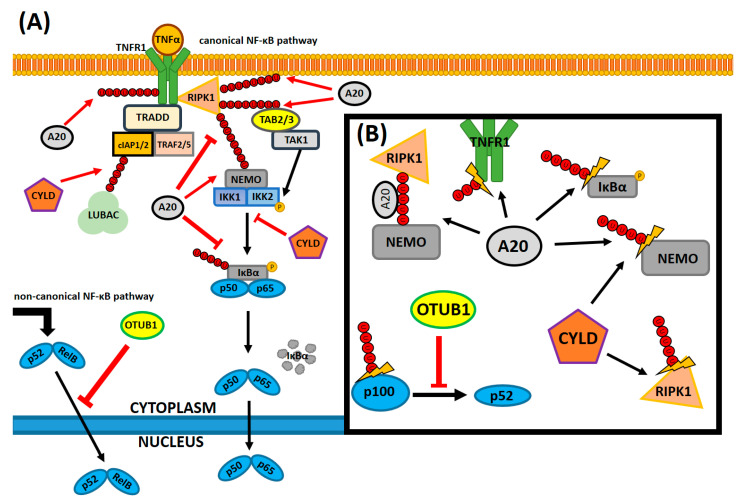
A simplified canonical NF-κB signaling pathway with its many deubiquitinating regulators and the molecular targets of those enzymes. Only a handful of all possible interactions between deubiquitinating enzymes (DUBs) and NF-κB pathway components are currently known and an even lesser amount of all the possible interactions are displayed here. (**A**) Canonical signaling in the NF-κB pathway consists of various receptors, where the signal is transduced through a cross-linked network of protein complexes and reaches the NF-κB heterodimeric subunits sequestrated in the cytosol. The signal from TNFα assembles the RIPK1 complex at the site of its binding with the receptor, which directs TAK1 to phosphorylate the IKK complex. In consequence, the IKK complex phosphorylates the inhibitor unit of sequestrated NF-κB proteins, which marks it for proteasomal degradation. After the proteolysis, the activated NF-κB heterodimer translocates to the nucleus and induces the transcription of target genes. Ubiquitin poly-chains, anchored by several amino acid residues (M1, K48 and K63, respectively, which, for the sake of the reading coherency, were not distinguished in this figure) to target proteins, fill a multitude of different roles: they create linkages and bridges between components and mark them for degradation or create additional signals. DUBs: A20 and CYLD are the key deubiquitinating regulators that not only remove ubiquitin chains from targets, but also bind to them. In addition, OTUB1’s novel role as an NF-κB inhibitor in the non-canonical pathway (not shown here). (**B**) Several Ub interactions of DUBs and the NF-κB effectors. A20 binds to M1-linked bridges between RIPK1 and NEMO, prohibiting other Ub-binding proteins from attachment and sequestrating the NF-κB-inducing signal. It also removes Ub chains from IκBα, thus disallowing its degradation and it replaces the Ub chain (from K63 to K48) on RIPK1, thus marking it for degradation. In addition, there is CYLD that competes with A20 on NEMO or RIPK1 degradation and affects several signaling components. Lastly, OTUB1 stabilizes p100, a progenitor protein of p52, which inhibits proteolytic degradation of p100 to 52, thus sequestrating the signal in the non-canonical NF-κB pathway. Based on [141,143,145,149]. Legend: black arrow—indicates non-antagonistic interaction or the direction of signal transduction in (**A**) or only points out the target protein in (**B**), red arrow—indicates antagonistic or competitive interaction, red arrow with a blunt end—indicates inhibition in (**A**,**B**), thunder-like symbol—indicates deubiquitination in (**B**). A20—tumor necrosis factor, alpha-induced protein 3, cIAP1/2—cellular inhibitor of apoptosis protein-1/2, CYLD—cylindromatosis tumor suppressor protein, IKK1/2—inhibitor of nuclear factor kappa B kinase alpha/beta, IKK1/2—inhibitor of nuclear factor kappa B kinase complex, IκBα—inhibitor of nuclear factor kappa B alpha, LUBAC—linear Ub chain assembly complex, NEMO—inhibitor of nuclear factor kappa-B kinase subunit gamma, NF-κB—nuclear factor kappa-light-chain-enhancer of activated B cells, OTUB1—ubiquitin thioesterase otubain-1, P—an indication of a protein-specific phosphorylation, p50—nuclear factor NF-kappa-B p105 subunit, p52—nuclear factor NF-kappa-B p100 subunit, p65 (RelA)—v-rel avian reticuloendotheliosis viral oncogene homolog A, RelB—v-rel avian reticuloendotheliosis viral oncogene homolog B, RIPK1—receptor-interacting Ser-Thr protein kinase 1, TAB2/3—TGF-beta-activated kinase 1, TAK1—mitogen-activated protein kinase kinase kinase 7, TNFR1—TNF receptor 1, TNFα—tumor necrosis factor α, TRADD—tumor necrosis factor receptor type 1-associated DEATH domain protein, TRAF2/5—TNF receptor-associated factor 2 and 5, Ub—ubiquitin.

A20 is the best-studied NF-κB-modulating DUB so far, however, it is important to mention another DUB regulator of NF-κB, the cylindromatosis tumor suppressor protein, CYLD [142]. The catalytic domain of CYLD, the ubiquitin specific protease domain (USP), is structurally also a cysteine protease, responsible for its DUB activity. CYLD, alongside A20, is suspected to remove lysine63-linked polyubiquitin chains from many targets involved in the transduction of the NF-κB signal, primarily NEMO and RIPK1 proteins [143]. CYLD exhibits affinity to different targets by binding adaptor proteins, e.g., p62, which guides it to the ubiquitinated ligands of interest [144]. Harhaj and Dixit described CYLD in a more investigative manner in the following articles [145,146]. OTULIN is a DUB that structurally solely constitutes the OTU domain with an N-terminal PUB-interacting motif, which is selective for binding methionine1-linked Ub chains in NF-κB transmitters [143,147]. Its systematic deficiency in expression causes OTULIN-related autoinflammatory syndrome (ORAS), which results in steatosis-alike liver inflammation [148]. However, not much is known about OTULIN and ORAS, as this protein functionality is still poorly understood and requires further investigations. Graphical interpretation of the DUBs functionality in modulating the NF-κB pathway and known interactions has been displayed in the Figure 2.

## 6. NF-κB Activity Research in Alcohol-Related Liver Injury Animal Models

To understand the properties and inducible transcription functionality of NF-κB, it is necessary to further ascertain the influence of this regulatory complex on the inflammatory responses of different liver cell types. Therefore, in this chapter, we describe the recent study reports that exposed various experimental and methodological approaches in regard to enlarging the current knowledge standing about NF-κB in relation to ALD.

### 6.1. The Potential Treatment Approach for Alcohol-Induced Liver Inflammation

Due to the lack of effective, pharmacological treatment approaches for advanced, severe ALD, it is important to improve the current medication and ongoing therapies addressing the disease progress and outcomes of ALD patients. As described in Section 3.1, in late-stage ALD, liver transplantation seems to be the conclusive medical solution [150]. The approach can be supported with steroid therapy, according to established Lille score guidelines [151] and in consonance with other statistical ALD-related models, e.g., Model for End-stage Liver Disease or Glasgow score [152]. However, only a complete withdrawal from alcohol consumption, proper psychological support and a healthier lifestyle are deemed proper ALD treatments. Otherwise, there are no further currently known options to fasten the regression of the pathological alcohol-induced liver condition. Therefore, reaching for novel therapeutics is a potent and viable strategy that can be supported by examining and uncovering the mechanistic aspects of the alcohol influence on NF-κB activation and its control. One of the groups of most promising sources of new active substances for medications is a group naturally derived plant extracts, already widely known and deeply rooted within the ancient Asian natural medicine and culture [153]. Nevertheless, semi-synthetic compounds like chloromethiazole (CMZ) [154] or microbial-derived solutions and pigments [155,156], fungi polysaccharides [157], carboxylic acids [158] and animal-derived byproducts destined for consumption [159] likewise have been taken under consideration. The target of these approaches is to measure the influence of diverse active substances on NF-κB functionality during variously conditioned alcohol-induced liver injuries and/or under inflammatory conditions, i.e., upon LPS stimulation [154,155,156,160,161]. The aim of the different experimental approaches here is to mimic the natural progression of ALD in chronic or acute-to-chronic alcohol intake and simulate the induction of a constant inflammatory liver state, followed up with the experimental attempts to demonstrate and examine particular stages of ALD, as described in Section 3.1, i.e., hepatic fibrosis [155,162]. The majority of compounds described here are a priori established as promising agents for candidate drugs in other diseases, mostly cancer, e.g., fucoxanthin [163], a pigment compound from a subset group of carotenoids, or piceid [164], a structural derivative of resveratrol and anticancer and anti-inflammatory stilbenoid [165]. The research focuses on potentially protective and efficient therapeutic factors, which modulate liver inflammation induced by either EtOH, its toxic metabolites or inflammatory stimulants. All of the reviewed studies were performed with animal models, with a majority compromising mice studies over rat model investigations, which were applied in [154,155,159,166,167]. After the in vivo experiments with different experimental setups, the animal liver samples were examined and, in most cases, the levels of NF-κB activity indicators as well as the signs of inflammation were assessed. In vitro studies were also conducted simultaneously with in vivo investigations [161] or performed exclusively [155,160], with rats and mice serving principally as primary cell type donors. Following the described aims, the exemplary fucoxanthin study in a mice model with a “prolonged acute” (feeding time of 7 days) alcohol feeding pattern showed alleviated levels of LPS/TLR4-induced NF-κB pathway activity in the fucoxanthin-pre-fed mice group, indicating its inhibitory influence on TLR4 [163]. The possible mechanism is discussed later in the chapter. The piceid (polydatin) research showed its hepatoprotective properties in the acute alcohol mice model by decreased NF-κB and metalloproteases activity in the pretreated group, resulting in a milder inflammatory state [164]. However, the mechanism of action is not fully understood and it requires further investigation, although the piceid-derived pro-hepatic antioxidant properties might be the key [164]. Several studies have been dedicated to research the influence of ginsenosides, natural steroids from the *Panax* plants (especially *Panax ginseng*), a well-known and widely applied natural plant group in traditional Chinese medicine [165]. As an example, rats [168] and mice [169,170] were fed with a ginsenosides-enriched diet, followed by alcohol intoxication in either prolonged acute/binge (9 days) [169] or chronic (6 weeks [170] and 12 weeks [168]) drinking settings. Binge mice pre-fed with ginsenoside Rg1 exhibited a higher survival rate after consecutive alcohol intoxication, whilst showing decreased production of pro-inflammatory cytokines (TNF-α, IL-1β and IL-6) and lowered NF-κB activity in a dose-dependent manner [169]. The possible anti-inflammatory mechanism may lie underneath the interaction between ginsenoside Rg1 and the glucocorticoid receptor, which after activation modulates NF-κB activity by functionally competing with the p65 subunit [171]. Another group with a pre-fed ginsenoside Rg1 chronic 12 (weeks) rat model reported similar anti-inflammatory properties of this compound with a noticeable decrease in NF-κB activity, along with diminished P450 2E1 expression, which resulted in lowered ROS cellular production [168]. The hepatocytes structure from ginsenoside Rg1 animals was also in better condition compared to non-treated controls, as alcohol is known to visibly damage the hepatocytes cytoskeletal network [172]. Lastly, the chronic setting was applied in a mice model treated with ginsenoside Rk3, the less active compound than Rg1 from *Panax ginseng*. The results were similar to previous ginsenoside treatments, with decreased pro-inflammatory cytokines production and reduced NF-κB activity [170]. The anti-inflammatory properties of Rk3 might be connected to its ability to induce the expression levels of GSH and superoxide dismutase (SOD, an enzyme-eliminating hydrogen peroxide [173]), thus alleviating the ROS-inflicted damage. Andrographolide, a diterpenoid isolated from *Andrographis paniculate*, appears to also negatively influence NF-κB activity in mice after 12 weeks of chronic EtOH feeding, however, the exact details about the mechanism are unknown [174].

Another set of experiments relied on pretreatment with microorganism-derived compounds, without animal EtOH intoxication. Secondary metabolites from *Monascus purpureus*, yellow pigments ankaflavin and monascin, known widely by their cytotoxic properties [175], showed a pro-apoptotic influence on HSC-T6 cells, but no influence on primary hepatocytes isolated from the liver of male Sprague-Dawley rats [155]. Those pigments seem to reduce proliferation of HSCs by invoking the G1 phase arrest, while inducing expression of pro-apoptotic genes, e.g., p53 and caspase-3, and decreasing NF-κB activity and thus reducing inflammation [155]. These data suggest a possible application of the yellow *Monascus*-derived pigments in treating hepatic fibrosis, however, the exact mechanisms of action are not yet fully understood. Another in vitro study on HSC-T6 utilized dioscin incubation and in vivo dioscin treatment in fibrosis-induced mice models [160]. Dioscin is a natural saponin, frequently used in natural medicine [176], and the study underlined its antifibrogenic property due to its inhibition of the TLR4/MyD88/NF-κB pathway. Furthermore, it was already mentioned in Section 3.3 that EtOH causes loosening of the gut endothelial cells tight junctions and increasing gut leakiness, resulting in free-roaming LPS concentrations and thus in microbial dysbiosis [177]. In another microorganism-based treatment study, Cui et al. observed that the culture supernatant from a probiotic microorganism species (e.g., *Lactobacillus reuteri* ZJ617, [178]) reduces the LPS-induced liver inflammation in mice by decreasing NF-κB activity. Additionally, the supernatant from *L. reuteri* is able to significantly raise the amount of expressed tight junction proteins (occludin, claudin 1, claudin 4, zonula occludens 1 (ZO1)), thus resulting in the loose gaps tightening and hence diminishing the LPS levels [156]. Interestingly, a similar result of enhanced expression of tight junction proteins was achieved with rice bran phenolic extract (RBPE) treatment, performed by another group [179]. In this study, mice underwent an 8-week EtOH-based liquid diet, supported with an RBPE solution mixture. The RBPE mice group exhibited lowered inflammation symptoms (including lower NF-κB activity) and the EtOH decrease in tight junction proteins was alleviated, in comparison with the non-RBPE control group. The EtOH-induced permeability of the intestinal barrier was reversed and it decreased the concentration of free-roaming LPS, which may explain the reduced activation rate of the LPS-TLR-NF-κB pathway [179]. As it was described previously in the text, oxidative stress plays a huge role in the progression of alcohol-related liver injury. Besides the aforementioned iNOS and COX-2 activities as indicators of enhanced cellular oxidative stress in the liver, the malondialdehyde (MDA) concentration increases with the progression of radical-driven unsaturated fatty acids oxidation [180]. In their paper, Song et al. pretreated Kunming mice for 3 weeks with alcohol and a melanin solution from the fungi *Lachnum* YM226, resulting in decreased NF-κB activity and increased antioxidant activity with enhanced GSH and SOD concentrations, as well as lowered COX-2 and iNOS in-liver activity [181]. Their work also helps with the structure identification of the melanin derived from *Lachnum* YM226. Zhao et al. applied a 5 week-long varied flavonoid treatment of EtOH-fed ICR mice, which concluded in noticeable alleviation of liver inflammation and NF-κB activity, as well as a significant reduction in COX-2 [182]. However, some of the applied flavonoids were more or less efficient in restoring particular functions, e.g., apigenin increased SOD and GSH levels, whilst naringenin reduced iNOS activity most efficiently. Due to the complexity of this paper and different results, we recommend the interested readership to refer directly to [182]. Radic et al. showed, in a study on a 6-week EtOH-fed chronic rat model, that whey can also reverse the EtOH-induced NF-κB activity levels in the liver. In addition, it was observed along with a noticeably inferior inflammation state and increased expression of glutathione peroxidase (GP) and SOD levels [159]. According to this article, the whey hepatoprotective influence may relate to its stimulation of GP expression. Green tea infusion after two weeks with a simultaneous EtOH diet in mice models seemed to alleviate the levels of MDA, iNOS and COX-2, and expression of NF-κB was also reduced [183].

Moreover, to further underscore the importance of antioxidant molecules, the molecular absence of a naturally occurring antioxidant named nuclear factor erythroid 2-related factor 2 (Nrf2) can lead to an increase in the pro-inflammatory cytokine production by enhanced stimulation of NF-κB [184]. Nrf2 is another transcription factor, however, it is responsible mainly for the regulation of expression of various antioxidant proteins [185]. Yan et al. reported that, after 5 days of a consecutive EtOH-based mice diet with cinnamic acid (CA) and syringic acid (SA), treatments increased levels of Nrf2 activity [158]. Additionally, CA and SA reversed the COX-2 and NF-κB activation enhanced by the EtOH diet. Interestingly, the fucoxanthin study also reported elevated levels of Nrf2 [163]. The study with an artichoke extract seemed to increase the SOD and GSH levels after ten days of consecutive EtOH feeding in mice, reducing the oxidative stress and suppressing NF-κB-driven liver inflammation [186]. Furthermore, the artichoke extract decreased the MDA concentrations, however, the hepatoprotective properties of artichoke or exact beneficiary compounds are unknown. Similarly, *Linderae radix* extracts also decreased MDA concentrations and reduced the inflammation severity, and cytochrome CYP2E1 expression was decreased [167]. It was not the only study where feeding/treating the animals with correspondent extracts provided the research groups with data and where decreased levels of metabolic oxidation by cytochromes (CYP21E, CYP1A, CYP4A10 and CYP4A14) were observed. The semi-synthetic compound chlormethiazole (CMZ) was applied as an inhibitor of CYP21E and thus concluded in a reduced LPS-induced inflammation level in Kupffer cells, isolated from Sprague-Dawley (SD) rats after 4 weeks of EtOH diet [154]. Schisantherin A decreased the EtOH-induced CYP2E1 and CYP1A2 expression levels in mice after 4 weeks of EtOH diet and suppressed NF-κB activity [187]. Interestingly, in this study, regarding the effects of schisantherin A on the liver cells, the main EtOH metabolic enzyme AHD activity decreased, while ALHD levels increased [187]. This might indicate the role of schisantherin A in supporting EtOH metabolism. The previously described piceid study also influenced CYP2E1 expression [164], and the CA and SA study also noted a decrease in CYP2EI expression [158]. Residing in the cytoplasm, protein aggregations called inflammasomes are responsible for inflammatory responses—one of them is known as nucleotide-binding domain, leucine-rich repeat (NLR) family, pyrin domain containing 3 (NLRP3). It is inducible by the activation of NF-κB transcription [188]. In the quercetin study, Wistar rats, for 17 days, underwent an EtOH-based diet along with quercetin treatment, which alleviated inflammation and induced expression of protective IL-10 [166]. Additionally, NRLP3 formation was proven to be diminished in the quercetin treatment study and was constrained by the expression of heme-oxygenase-1 (HO-1) [166]. HO-1 expression is diminished in patients with an acute liver injury [189] and quercetin seems to elevate its levels again. The exact mechanism is unknown, however, as the name suggests, it might rely on the role of iron metabolism in the body, which HO-1 mediates [189]. Eight-weeks feeding of rats with the Lieber–DeCarli model and umbelliferone treatment resulted also in increased IL-10 expression levels as in the quercetin paper, while pro-inflammatory cytokines and NF-κB activation decreased [162]. Umbelliferone seems to activate the antioxidant system, however, the exact mechanism is not fully elucidated. In addition, another transcriptional factor that crosstalks with NF-κB and cooperatively regulates inflammation, immune responses and cell proliferation is the signal transducer and activator of transcription 3 (STAT3) [190]. STAT3 activity levels were estimated to be inferior after the kahweol treatment, a diterpenoid derived from coffee beans, described in an in vitro study by Seo et al. [191]. This observation was reported in isolated Kupffer cells and hepatocytes that were incubated with kahweol extract and the inflammation was induced by LPS addition. Presumably, kahweol also decreased the NF-κB activity in those cells. An analogical situation has been observed for the mitogen-activated protein kinase (MAPK)-induced pathway that can induce NF-κB activity as well [192]. Its levels were noticeably decreased as well after kahweol treatment [191] and an *L. reuteri* study [156]. Lastly, a decreased lipid accumulation effect was observed through increased levels of AMP-activated protein kinase (AMPK) and peroxisome proliferator–activated receptor α (PPARα), which is a physiological NF-κB inhibitor [193]. The polysaccharide peptide (PSP) of ALD mice from *Coriolus versicolor* reduced the EtOH-induced inflammation by enhancing AMPK activity levels and thus suppressing NF-κB activity [157]. Similarly, the enhanced AMPK and (PPAR)-α levels compared to controls were noted in Hsu et al., where aqueous extract from pepino was applied to Lieber–DeCarli diet-fed mice after a 5-week experimental setting [194]. Importantly, also decreased lipid accumulation was observed by diminished activity levels of a lipogenic enzyme, called *sterol regulatory element-binding protein* 1 (SREBP-1), which during chronic liver inflammation supports lipogenesis [195]. SREBP-1 activity was suppressed as well during the treatment with acanthotic acid by Song et al., in an acute EtOH setting with mice models [158]. They also utilized the HSC-T6 cells with LPS stimulation and alcohol incubation. The major finding of this setting, however, is the decreased levels of lipin1/2, which are important regulators of lipid metabolism [196]. However, as in previous study cases before, the exact mechanisms of action require elucidation as well.

The summarization of key findings and experimental conclusions from this chapter are gathered and described in Table 1. To support the readership with a better understanding of the provided descriptions of studies gathered in this chapter, it is worth to mention and introduce the so-called liver function tests [197]. These tests are often used to assess the severity of liver damage and on-going stage of liver disease. The most popular one, however, is the transaminases test, where the ratio of specific transaminases (aspartate and alanine transaminase, AST and ALT, respectively) is evaluated from blood serum samples [197]. The AST/ALT ratio in ALD patients is frequently higher than 1 [197]. Although not specified before in the text, a magnitude of the treatment studies already applied this test to blood samples harvested from animal models. The goal was to evaluate the levels of EtOH-related liver injury, indicating the applied ALD-like condition after EtOH feeding, whilst most of the active compound treatments restored the original AST/ALT ratio back to below 1.

### 6.2. Genetic Manipulations

An abundance of papers in the field of NF-κB and ALD involved gene manipulation approaches, where certain target genes were turned inoperative or overly operative. The achievement of such results is possible through multiple genetic engineering modifications of target genes, including siRNA genes repression, gene knockouts (KOs), gene transfection, gene deletion and gene recombination, conducted in animal and in vitro models [198,199]. The application of these techniques aims to establish the molecular mechanism principles behind the aforementioned complex functionality in alcohol-related liver disease. To assess the role of Kupffer cells in ALD progression, Maraslioglu et al. extracted liver samples from recombinant *cis*-NF-κB^EGFP^ (enhanced green fluorescent protein—EGFP) mice after 4 weeks of chronic EtOH feeding and observed increased levels of pro-inflammatory cytokines production (IL-6, TNF-*α*) due to NF-κB upregulation [200]. To visualize EGFP-modified NF-κB activity induction, the epifluorescence microscopy technique was applied. They also challenged KC with LPS stimulation in vitro. Further evidence in EtOH-induced NF-κB activation was acquired by knockouts of pre-mRNA-splicing factor SLU7 by Wang et al. in a chronic-plus-binge mouse model [201]. After 10 days of consecutive EtOH feeding, the mice were challenged with a high dose of alcohol and sacrificed afterwards. The knockout of SLU7 resulted in increased expression of sirtuin 1 (SIRT1), which acts as a response to pro-inflammatory stimuli [202] and the previously mentioned lipin-1. SLU7 deficiency also caused decreased NF-κB activation, which suggests its role as a mediator in the inflammatory liver condition [201]. Following this approach, from the other side, the direct lipin-1 KO in myeloid cells during the ALD condition (Gao-binge ethanol feeding pattern, mouse model) resulted in the mitigation of the pro-inflammatory liver state [203]. Primarily due to reduced NF-κB activity, however, lipin-1-deficient mice expressed higher levels adiponectin, thus handled lipid metabolism more efficiently than control groups. The possible mechanism may relate to the lipid-mediating adiponectin-ileum-derived fibroblast growth factor 15 (FGF15), with this also being stimulated in this research study [203]. Furthermore, the importance of those factors seems to be indeed relevant for ALD development and NF-κB activity, as a KO study of the mitochondrial outer membrane-anchored MitoNEET (mNT) protein provided further interesting data [204]. MitoNEET is suspected to be a component of mitochondrial redox reactions in the respiration chain [205]. In this study, Hu et al. observed increased levels of adiponectin, SIRT1 and FGF15 in a Lieber–DeCarli mouse model after 4 weeks of treatment, while NF-κB signaling was diminished compared to controls [204]. Unfortunately, the exact mechanism of action is, to our current knowledge, not yet fully understood. Another interesting research regarded the activity of the interleukin-1 receptor-associated kinase M (IRAKM), which appears to play a significant role in anti-inflammatory responses by the “second activation” of NF-κB transcription [206]. In this setting, the KO of IRAKM in mice chronically (3 weeks) fed with EtOH in a dose-dependent manner, after a challenge with low-dose LPS, resulted in the formation of IRAKM Myddosome, a MyD88-dependent NF-κB-mediating complex [207]. This complex mediates also the expression of macrophage-inducible C-type lectin (Mincle) in macrophages, an important cell death detector [208]. It was also proven that hepatocytes incubation with EtOH increased the release of ligand spliceosome-associated protein 130 (SAP130), the Mincle-associated substrate [208]. The exact details on those compartments’ involvement in ALD development require further elucidation. To support the previous mention about the importance of iron management in liver tissue, Zmijewski et al. showed, in Lieber-DeCarli mouse models, decreased levels of siRNA of hepcidin, the iron-regulating hormone [209]. Interestingly enough, the KO of TLR4 seems to alleviate this effect, however, the mechanistic details are unknown. The repression of the Axl gene (a member of the tyrosine kinases receptor family) in HSCs and its activator ligand, the vitamin K-dependent product of growth arrest-specific gene 6 (Gas6), resulted in decreased activation of HSCs, which supports the involvement of Gas6 in the NF-κB pathway [210]. In other words, increased levels of Axl and its ligand accompany the progression of ALD, however, in this experiment, liver fibrosis was induced in mice models by tetrachloromethane (CCl_4_), not EtOH feeding. Nevertheless, we decided to cover this review in regard to curious insights on the role of HSCs in ALD development, which is still very enigmatic to this date. The group of Lee et al. tested the possible role of the novel interleukin 32 (IL-32), which is suspected to be an important factor in carcinogenesis [211]. IL-32 overexpression decreased the activity of NF-κB and the expression rate of inflammatory cytokines (IL-6) after 6 weeks of treatment in a Lieber–DeCarli mice model, as well as inhibiting cytochrome P450 2E1 expression and thus reducing oxidative stress. COX-2 activity was significantly reduced [212]. In the C-X-C motif chemokine 11 (Cxcl11) gene repression studies, mice were fed with EtOH and a high-fat diet chronically for 3 months [213]. This chemokine apparently mediates the synergistical (yet harmful) effects between EtOH and a high-fat diet in the liver that lead to enhanced neutrophil infiltration in the liver, and thus a more severe inflammation condition, compared to controls. However, the exact mechanism of mediation has still not been covered yet [213]. Moreover, the protein tyrosine phosphatase 1B (PTP1B) is an important mediator of inflammation in hepatic fibrosis by inducing the activation of HSCs [214]. Chen et al. showed elevated PTP1B levels in CCl_4_-induced fibrotic Kungming mice, which might underline its role as a fibrogenesis stimulant [214]. Interestingly, the siRNA silencing of PTP1B decreased the levels of IL-6 and TNF-α, thus diminishing NF-κB activity [214], which even further supports PTP1B’s role as a possible pro-inflammatory regulator [215]. Lastly, the group of Chao et al. proved the importance of transcription factor EB (TFEB) in ALD development [216], which acts as the regulator of lysosomes formation [217]. In their study, mice with TFEB deficiency or mice with adenoviral-driven overexpression of TFEB were given EtOH in the acute setting to induce EtOH-related liver damage [216]. It turned out that alcohol intake suppresses the level of TFEB expression, while TFEB overexpression led to a less severe inflammation state and increased lysosomal biogenesis, compared to controls. However, the exact mechanism of action has not been covered, and the mechanism of alcohol-impairing lysosomal functions is unknown too [216].

The detailed analysis, experimental settings and primary resolutions of these findings with corresponding references are depicted, in essence, in Table 2.

### 6.3. Further Studies

Lastly, we surveyed the papers that included miscellaneous experimental approaches to assess the NF-κB role in ALD. An interesting study in regard to the estrogen correlation with alcohol consumption involves female mice models and beer drinking—there is evidence that hinted the possibility of estrogen loosening gut tight junctions and increasing circulating LPS levels [218]. On the other hand, beer in this study apparently causes lesser inflammation compared to EtOH in the same dose, which suggests its hepatoprotective role [219]. Furthermore, the cells tend to release a repertoire of bi-layered membrane vesicles known as extracellular vesicles (EV) that gathered, in recent years, elevated interest from the research community [220]. Interestingly, small fragments of RNA known as microRNA (miRNA) are often derived from EVs and are often associated with liver diseases progression [221]. EVs can be perceived as detectable barcodes and thus a study was conducted to investigate their release levels from cells in a simulated ALD condition, and their miRNA levels were measured. The increased levels of blood EVs in the early alcoholic steatohepatitis rat models were detected and the upregulated miRNA analyses showed over 120 potential target genes (including, e.g., Wnt proteins, important signal transductors [222]) involved in inflammatory and cancer pathways, while three of them (let7f, miR-29a and miR-340) were significantly raised in ALD mice than in control groups [223]. Our group performed a similar study, where increased miRNA levels of miR-122 and let7f were observed in the blood samples of alcoholic patients [224]. This gives another indication that, besides standard liver function tests, EVs have potency to become indicators of hepatocytes damage in alcohol-driven liver injury. The HSCs stimulation by *E. coli* RNA concluded in stimulating the NLRP3 inflammasome that hints the role of this complex in hepatic fibrosis progression [225]. Furthermore, this evidence is supported by a previously described study with quercetin, which showed lowered formation of this complex [166]. Another group focused on the Kupffer cells polarization telomerase reverse transcriptase (TERT) and its role in the feedback loop signaling of NF-κB, and the acquired data exhibit a tendency of TERT upregulation by alcohol intake, thus leading to a conclusion that TERT may exhibit a particular role in macrophage activation during inflammation. Additionally, in the same study, applying an NF-κB inhibitor, a compound named pyrrolidine dithiocarbamate (PDTC) [226], resulted in repression of TERT activity, hence leading to a conclusion that there is a correlation between KC activation, alcohol-mediated inflammation, NF-κB signaling and the aforementioned transcriptase [227]. The inhibition of spleen tyrosine kinase by the novel orally administrated SYK inhibitor, R406 [228], responded in the functional activity diminishment of neutrophil infiltration and decreased NF-κB activation, along with limited inflammasome formation, thus hinting SYK to be a potential [229]. The last sort of publications comprise human-based studies where levels of different ALD molecular indicators, such as steroid sulfatase (STS) [230], inflammatory stimuli, the β-klotho enzyme or hormonal fibroblast growth factors (FGFx), were experimentally estimated [231,232,233]. In addition, supporting the previously described estrogen influence with the correlation of alcohol consumption, an experiment to measure STS levels was performed on patient-derived serum samples [231]. Therefore, there is evidence that estrogen enhances the negative side effects during alcohol intoxication [230], thus it probably further suggests why females suffer more drastically from binge drinking effects [234]. Described in this section, the remaining works are collectively gathered in Table 3, altogether with all the necessary details and highlighted primary findings in regard to the subject. In concluding this review, the following section displays several experimental approaches and juxtaposed data from different studies, conducted by various groups. Consequently, plentiful potential therapeutic targets, research directions and uncovered mechanistic aspects in regard to NF-κB and alcohol-mediated inflammatory disease have emerged. The possible discussion related to the future studies revolving around the subject are contained in the following final section of this review.

## 7. Discussion

The importance of ALD and other alcohol-influenced medical conditions cannot be neglected, as chronic and acute alcohol consumption remains a grave problem worldwide. Alcohol drinking is a source of excessive social and individual harm that culminates in higher mortality rates [237]. The innate immune response and the entire systematic inflammation represent a mechanistically complicated and convoluted molecular apparatus that still requires scientific effort to be fully understood. The current state of the art in medicine is still limited in terms of treating advanced ALD and its consequences, as liver transplantation is ultimate and sometimes the sole treatment option in severe causes. It is important to seek new methods and novel potential therapies and further studies in the field of the alcohol impact shall be conducted in the future. Furthermore, the crosstalk between NF-κB and other significant signaling pathways is still poorly understood, as the formation of inflammasomes and signaling ligand–receptor interactions are complex topics. Although the assessment of the role of alcohol and the prevention of alcohol-related diseases could fasten the research acceleration in that matter, it should be highly advised for multi-layered studies to be designed and executed. As alcohol “travels” abundantly through the human body and has an impact not only on the liver, but also crosses the brain–blood barrier (BBB), the focused cooperative studies on that matter should be highly beneficial. Given that lots of novel potentially hepatoprotective substances are being currently investigated, there are almost no known compounds that could exhibit the same properties for brain cells, which in particular get damaged by accumulation of MeCHO. Since its discovery, the NF-κB complex has been heavily investigated and it is associated with almost any kind of inflammatory process. As inflammation is a broad immune response by a multitude of cells, connected with a vast network of signaling pathways and signalers, NF-κB still constitutes a hallmark for this process. The key to improve and enhance the efficiency of ALD studies is the proper application of future experimental settings that utilize proper NF-κB activity measurements. More precise EtOH dose-dependent, time-dependent and manner-dependent studies are required, as, despite all the invaluable animal models we possess, there is still no perfect setting for either chronic or acute EtOH research. Especially since there are significant differences between species’ inner metabolism, the conditions that are applied in mouse settings do not have to apply to humans [238]. This raises a question on the necessity of introducing more human studies to the research, however, chronic EtOH studies that follow patterns of alcoholism development are almost impossible to perform in human models. Significantly, to overcome those obstacles, the focus should be put on molecular basic research and understanding the still not elucidated molecular factors, crosstalks with other pathways and, most importantly, different regulators. Interestingly, in Huang et al., the levels of the A20 protein were diminished in lung macrophages isolated from alcoholic mice models in the chronic setting, while the levels of pro-inflammatory cytokines increased [239]. This suggests an important role of A20 in the inhibition of the inflammation in alcohol-induced organ damage, however, further experimental evidence is necessary to proceed. Our group will assign a special interest to DUBs in the future, and we truly believe that their role in the control of NF-κB and thus ALD development is indeed enormous. More studies on the topic, mixing together active compounds, cellular studies and genetic approaches, must be conducted in order to uncover what is yet to be covered.

As far as alcohol drinking has been an inseparable part of humanity and human societies for millennia, as long as we progress scientifically, alcohol drinking and its issues should also progress and become less perilous. In the end, most of our culture is built upon alcohol drinking and this issue touches us all.

## Figures and Tables

**Table 1:** A summary overview of research studies indicating the protective/therapeutic influence of various compounds in alcoholic liver disease (ALD). **↑** —indicates an increase, **↓**—indicates a decrease. ADH—alcohol dehydrogenase, AH—alcoholic hepatitis, ALD—alcoholic liver disease, ALDH—aldehyde dehydrogenase, ALI—alcohol-related liver injury, ALT—alanine transaminase, AMPK—adenosine monophosphate-activated protein kinase, AST—aspartate transaminase, C57BL/6—C57 black 6, CMZ—chlormethiazole, COX-2—cyclooxygenase 2, CYP2E1/1A2—cytochrome 2E1 and 1A2, EtOH—ethanol, GP—glutathione peroxidase, GSH—glutathione, HO-1—heme-oxygenase-1, HSCs—hepatic stellate cells, ICR—Institute of Cancer Research, IL—interleukin, iNOS—nitric oxide synthase, IκB—inhibitor of kappa B, LPS—lipopolysaccharide, MAPK—mitogen-activated protein kinase, MDA—malondialdehyde, MyD88—myeloid differentiation primary response 88 protein, NF-κB—nuclear factor kappa-light-chain-enhancer of activated B cells, NLRP3—nucleotide-binding domain, leucine-rich repeat (NLR) family, pyrin domain containing 3, Nrf2—nuclear factor erythroid 2-related factor 2, p53—tumor suppressor p53, p65 (RelA)—v-rel avian reticuloendotheliosis viral oncogene homolog A, PPARα—peroxisome proliferator–activated receptor alpha, RBPE—rice bran phenolic extract, SOD—superoxide dismutase, SPF—specific pathogen-free, SREBP-1c—sterol regulatory element-binding protein, STAT3—signal transducer and activator of transcription 3, TLR2/4—toll like receptor 2 and 4, TNFα—tumor necrosis factor alpha, ZO-1—zonula occludens-1.

Type of Study	Model Organism/Isolation Source	Cell Type/Cell Line/Tissue	Tested Substance(S), Derivative(S), Compound(s)	Compound(s) Source(s)	Conditions	Primary Findings/Results	Ref.
In vivo	SD rats	Kupffer cells	CMZ	semi-synthetic	ALD, LPS stimulation	↓ levels of CYP2E1, ↓ accumulation of NF-κB p65 subunit and TNF-α	[154]
In vitro	SD rats	HSCs/ HSC-T6, primary hepatocytes	ankaflavin, monascin	*Monascus purpureus,* fermentation	ALD	↑ levels of p53, ↑ caspase 3 activity, ↓ levels of NF-κB expression, ↑ levels of IκB expression	[155]
In vivo	C57BL/6 mice	blood, liver tissue	culture supernatant	*Lactobacillus reuteri* ZJ617, *Lactobacillus rhamnosus* GG	ALD, LPS stimulation	↓ levels of serum ALT and AST, ↑ levels of claudin 3, ZO-1 and occludin, ↓ IL-6 and TNFα, ↑ expression of IL-10, ↓ expression of TLR4, ↓ NF-κB, ZJ617s suppress TLR4/MAPK/NF-*κ*B activation	[156]
In vivo	C57BL/6J mice	blood, liver tissue	polysaccharide peptide (PSP)	*Coriolus* *versicolor* JNPF-CV05 strain	ALD, chronic and binge models	↓ ALT, AST and MDA, ↑ activity of AMPK and PPARα, ↓ levels of TLR2, TLR4 and NF-κB	[157]
In vivo	BALB/cA mice	liver tissue	cinnamic acid, syringic acid	*-*	ALD, ALI	↓ levels of CYP2E1, COX-2 and NF-κB, ↑ Nrf2 expression, ↓ levels of IL-6 and TNFα	[158]
In vivo	Wistar rats	liver tissue	whey	-	ALD, ALI	↑ SOD and NF-kB protein levels, lower inflammation after whey consumption	[159]
In vivo	ICR mice	liver tissue	fucoxanthin	marine seaweed	ALD, ALI	↑ expression of Nrf2-mediated signaling pathway, ↓ TLR4 and NF-κB	[163]
In vivo	C57BL/6 mice	blood, liver tissue	polydatin (piceid)	*Picea sitchensis*	ALD, ALI	↓ levels of serum ALT and AST, ↓ expression of CYP2E1, ↓ of NF-κB	[164]
In vivo	SPF-Wistar rats	blood, liver samples	quercetin	-	ALD, AH, acute ALI model	↑ HO-1, ↓ NLRP3, ↓ activity of NF-κB, ↑ promotion of IL-10	[166]
In vivo	SD rats	blood, liver tissue	linderae radix	*Lindera aggregata*	ALD, ALI	↓ levels of serum ALT, AST, MDA, ↓ level of CYP2E1, ↓ NF-κB, TNF-α and IL-1β	[167]
In vivo	SD rats	blood, liver tissue	ginsenoside Rk3	*Panax ginseng*	ALD, chronic drinking	↓ levels of caspase-3 and caspase-8, ↓ levels of CYP2E1 expression, ↓ levels of NF-κB	[168]
In vivo	C57BL/6 mice	blood, liver tissue	ginsenoside Rg1	*Panax ginseng* C.A. Mayer	ALD, ALI, binge drinking	↓ levels of hepatic TNF-α, IL-1β and IL-6, ↓ levels of NF-κB activity, ↑ levels of glucocorticoid receptor, ↓ levels of ALT and AST	[169]
In vivo	C57BL/6 mice	blood, liver tissue	ginsenoside Rg1	*Panax ginseng*	ALD, chronic drinking	↓ levels of NF-κB, ↓ production of TNF-α, IL-6 and IL-1β, ↑ expression levels of SOD and GSH	[170]
In vivo	C57BL/6J	blood, liver tissue	andrographolide	*Andrographis paniculata*	ALD, ALI	↓ the hepatic levels of NF-κB and TNFα, ↓ levels of serum ALT, AST,	[174]
In vivo	C57BL/6 mice	liver tissue	RBPE	*Oryza sativa*	ALD, ALI	↓ expression of ZO-1, claudin-1 and claudin-4, ↓ microbiota dysbiosis, attenuated activation of LPS/TLR4/NF-κB pathway	[179]
In vivo, in vitro	Kunming mice	liver tissue, HepG2	melanin	*Lachnum* YM226	ALD, ALI	↓ hepatic levels of NF-κB, IL-6 and TNFα, ↓ hepatic activities of iNOS and COX-2	[181]
In vivo	ICR mice	liver tissue	apigenin, quercetin, naringenin, (−)-epigallocatechin gallate, genistein	flavonoids	ALD, ALI	genistein mitigates fibrosis and naringenin mitigates apoptosis, ↓ levels of NF-κB p65, COX-2 and IL-6, ↓ serum levels of AST, ALT	[182]
In vivo	C57BL/6 mice	blood, liver tissue	green tea infusion	*Camellia sinensis*	ALD, ALI	↓ levels of serum ALT, AST, MDA, ↓ expression of TLR4 and NF-κB, ↓ expression of iNOS	[183]
In vivo	ICR mice	blood, liver tissue	artichoke extract	*Cynara scolymus L.*	ALD, ALI	↓ levels of serum ALT, AST, MDA, ↓ expression of TLR4 and NF-κB	[186]
In vivo	C57BL/6	blood, liver tissue	schisantherin A	*Schisandra chinensis*	ALD, ALI	↓ levels of serum ALT and AST, ↓ CYP2E1 and CYP1A2 expression, ↓ NF-κB, ↓ ADH, ↑ ALDH	[187]
In vivo	C57BL/6 mice	blood, liver tissue	aqueous extract	Pepino *(Solanum muriactum)*	ALD, Lieber–DeCarli diet	↓ serum levels of AST and ALT, ↑ AMPK and PPAR-α, ↓ SREBP-1c, ↓ TNFα and IL-6, ↓ activity of NF-κB	[194]
In vitro	mice	HSCs/HSC-T6, primary hepatocytes	dioscin	-	ALD, LPS stimulation	↓ levels of MyD88, NF-κB, IL-1, IL-6, TNFα, TLR4, expression	[160]
In vivo, in vitro	C57BL/6	HSC-T6, liver tissue	acanthoic acid	*Annona amazonica*	lipogenesis model, LPS stimulation	↓ expression of SREBP-1, and lipin1/2, ↓ fat droplets caused by EtOH/LPS. ↓ expression of TLR4 and NF-κB	[164]
In vivo	C57BL/6 mice	Kupffer cells, hepatocytes	kahweol	coffee beans	ALD, LPS stimulation	↓ levels of IL-1α, IL-1β, IL-6 and TNFα, ↓ STAT3 and MAPK, ↓ activation of NF-κB	[191]
In vivo	SD rats	liver tissue	umbelliferone (7-hydroxycoumarin)	*Umbelliferae* plant family	ALD, fibrosis, Lieber–DeCarli	↓ levels of TNF-α and IL-6, ↑ levels of IL-10, ↓ levels of TLR4 and NF-κB, improved mild hepatic fibrosis	[162]

**Table 2:** A summary overview of genetic engineered-based studies in the field of ALD research. **↑**—indicates an increase, **↓**—indicates a decrease. ALD—alcoholic liver disease, ALI—alcohol-related liver injury, ALT—alanine transaminase, AST—aspartate transaminase, Axl—tyrosine-protein kinase receptor UFO, COX-2—cyclooxygenase 2, cxcl11—C-X-C motif chemokine 11, EGFP—enhanced green fluorescent protein, ER—reporter of expression, EtOH—ethanol, FGF15—fibroblast growth factor 15, Gas6—growth-arrest-specific gene-6, HCs—hepatocytes, HFD—high-fat diet, HSCs—hepatic stellate cells, IL—interleukin, IRAKM—interleukin-1 receptor-associated kinase M, KO—knockout, LPS—lipopolysaccharide, Mincle—macrophage inducible Ca^2+^-dependent lectin receptor, SIRT1—NAD-dependent deacetylase sirtuin-1, mNT—mitochondrial outer membrane-anchored MitoNEET, NF-κB—nuclear factor kappa-light-chain-enhancer of activated B cells, OE—overexpression, p50—nuclear factor NF-kappa-B p105 subunit, p65 (RelA)—v-rel avian reticuloendotheliosis viral oncogene homolog A, PTP1B—the protein tyrosine phosphatase 1B, SAP130—spliceosome-associated protein 130, SH—steatohepatitis, siRNA—small interfering RNA, SLU7—pre-mRNA-splicing factor SLU7, TFEB—transcription factor EB, TLR4—toll like receptor 4, TNF-α—tumor necrosis factor alpha.

Type of Study	Model Organism, Isolation Source	Cell Type/Cell Line	Target, Method	Condition	Primary Findings/Results	Ref.
In vivo	mice	HSCs	Gas6/Axl, siRNA silencing	ALD, ALI, fibrosis	↑ serum levels of Gas6 and Axl with chronic disease progression, Gas6/Axl compulsory for HSCs activation	[210]
In vivo	mice	HCs	TFEB, deletion and OE	ALD, ALI	overexpression of TFEB led to ↓ of lysosomal biogenesis and mitochondrial activities, KO mice developed more severe ALI syndromes	[216]
In vivo, in vitro	mice	HCs from mice, HepG2 and Huh7 lines	IL-32γ, OE and transfection	ALD, ALI	↓ levels of COX-2 and IL-6, ↓ level of NF-*κ*B activity	[212]
In vivo	mice	Kupffer cells	NF-κB with EGFP, ER	ALD, ALI, LPS stimulation	LPS and chronic EtOH ↑ levels of NF-*κ*B activity, ↑ expression levels of IL-6 and TNF-𝛼	[200]
In vivo	mice	myeloid cells	lipin-1, deletion	ALD, ALI	↑ levels of adiponectin and FGF-15 expression, ↓ NF-κB activity	[203]
In vivo	mice	HCs	SLU7, KO	ALD, ALI	↑ expression levels of SIRT1 and lipin-1, thus ↓ NF-κB activity	[201]
In vivo	mice	HCs	mNT, KO	ALD, ALI, SH, Lieber–DeCarli	↓ levels of AST and ALT, ↑ levels of adiponectin and FGF-15 expression, ↑ of SIRT1 and ↓ NF-κB activity	[204]
In vivo, ex vivo	mice	HCs, bone marrow macrophages	IRAKM, KO	ALD, ALI, LPS stimulation	Mincle ligand SAP130 activates inflammatory response, LPS activates the formation of IRAKM Myddosome, IRAKM or Mincle deficiency protects from ALI, ↓ NF-κB activity	[207]
In vivo	mice	HCs	TLR4, KO	ALD, ALI, Lieber–DeCarli	↓ translocation of NF-κB p65, ↓ binding of NF-κB p50 to hepcidin, ↓ NF-κB activity	[209]
In vivo, in vitro	mice	liver cells	cxcl1, deletion	HFD, binge drinking, ALD	Cxcl11 deletion caused ↓ level of HFD + EtOH-related inflammatory response, overexpression of cxcl11 caused ↑ of SH syndrome,	[210]
In vivo, in vitro	mice	liver cells, macrophages	PTP1B, siRNA silencing, OE	ALD, ALI, LPS stimulation	silencing of PTP1B resulted in ↓ levels of IL-6 and TNF-α, while PTP1B overexpression led to ↑ inflammation, PTP1B can regulate NF-κB activity	[214]

**Table 3:** A summary of the remaining experimental approaches in the ALD-NF-κB-related investigation. **↑** —indicates an increase, **↓**—indicates a decrease. 4-HNE—4-Hydroxynonenal, AIN-93G—American Institution of Nutrition 1993 rodent diet, ALD—alcoholic liver disease, ALI—alcohol-related liver disease, ALT—alanine transaminase, ASH—alcoholic steatohepatitis, AST—aspartate transaminase, bcl-2—B cell lymphoma 2,C57BL/6—C57 black 6,CD14—cluster of differentiation 14 protein, *E. coli—Escherichia coli,* EGF—epidermal growth factor, EtOH—ethanol, EV—extracellular vesicles, FGF19/21—fibroblast growth factor 19/21, HCs—hepatocytes, HSCs—hepatic stellate cells, IFN—interferon, IL—interleukin, iNOS—nitric oxide synthase, IκBα—inhibitor of nuclear factor kappa B alpha, KC—Kupffer cells, let7f—the lethal-7 microRNA precursor, LPS—lipopolysaccharide, miR-29a/340—microRNA 29a/340, miRNA—microRNA, MyD88—myeloid differentiation primary response 88 protein, NF-κB—nuclear factor kappa-light-chain-enhancer of activated B cells, NLRP3—nucleotide-binding domain, leucine-rich repeat (NLR) family, pyrin domain containing 3, PAI-1—plasminogen activator inhibitor-1, PTDC—pyrrolidine dithiocarbamate, qRT-PCR—real-time quantitative PCR polymerase chain reaction, SD—Sprague-Dawley, STAT3—signal transducer and activator of transcription 3, STS—steroid sulfatase, SYK—spleen tyrosine kinase, TA—telomerase activity, TERT—telomerase reverse transcriptase, TGFβ1—transforming growth factor β1, TLR4/7—toll like receptor 4 and 7, TNFα—tumor necrosis factor alpha, Wnt—Wingless and Int-1 signaling pathway.

Type of Study	Model Organism, Isolation Source	Cell Type/Organ	Approach	Condition	Primary Findings/Results	Ref.
In vivo	Wistar rats	blood, liver cells	nucleotide-supplemented AIN-93G rodent diet	ALD, ALI	↓ serum levels of AST and ALT, ↓ plasma LPS and inflammatory cytokine levels, ↓ levels of TLR4 and CD14, ↓ phosphorylation of IκBα and NF-κB p65 in the liver	[235]
In vivo	SD rats	liver cells	EtOH-induced hepatic miRNA expression before/after partial hepatectomy	ALD, ALI	hepatic miRNAs expression pattern changes in chronic drinking rather than acute binging, after hepatectomy the miRNA expression changed in chronically alcohol-exposed liver	[223]
In vivo	ASH mice	blood, liver cells, HCs	miRNA (barcodes) in extracellular vesicles (EV) measurement	ALD, ASH, ALI	↑ expressed blood EV in early ASH, 9 ↑ and 4 ↓ miRNAs expression, 121 potential target genes incl. NF-κB, EGF, Wnt, and bcl2; let7f, miR-29a, and miR-340 were expressed in EVs from ASH mice	[236]
Human studies, in vitro	human	blood, liver cells	STS expression levels analysis	ALD, ASH, cirrhosis	↑ levels of circulating estrogens in patients’ serum, activation of NF-κB leads to STS expression	[231]
In vivo, in vitro	C57BL/6J female mice	blood, liver cells	Female mice beer (stout and pilsner) feeding	ALD, acute beer consumption, LPS stimulation	mRNA expression of SREBP1c stays the same between groups, ↑ levels of expression of MyD88, iNOS, 4-HNE adducts, NF-κB and PAI-1 in EtOH groups, not in the beer groups	[219]
Human studies, in vitro	human	blood, liver cells, Huh-7	FGF19, FGF21 and β-Klotho levels evaluation	ALD, ALI, ASH	in human samples ↑ expression levels of IL-1β, IL-6 and TNFα, ↓ levels of β-Klotho, in cell cultures ↑ levels of FGF21 and ↓ levels of β-Klotho levels	[232]
Human studies, in vitro	human	blood, liver cells, HCs	qRT-PCR in early ALD in human patients, in vitro TLR7-IFN pathway stimulation	ALD, ALI	↑ levels of IL-1β, TNFα and NF-κB in early ALD, ↓ levels of IL-6/STAT3 and cyclin D lead to ↓ proliferation and HCs apoptosis, ↑ activation of TLR7–IFN axis in HCs	[233]
In vitro	rat	HSCs (HSC-T6)	HSCs stimulation by *E. coli* RNA	ALD, ALI, fibrosis	↑ levels of IL-1β and TGF-β1 secretion by HSC-T6 after *E. coli* RNA stimulation, as well as ↑ expression of caspase-1, while ↓ procaspase-1, (TGF-β1) overproduction in HSC-T6, *E. coli* RNA can stimulate NLRP3 inflammasome activation	[225]
In vivo, in vitro	C57BL/6 mice	liver cells, KCs	The role of TERT in macrophage activation in ALD	ALD, ALI, LPS stimulation	↑ levels of TERT expression and TA in vivo, ↑ levels of TERT in vitro, an NF-κB inhibitor PDTC ↓ levels of TERT in macrophage polarization	[227]
In vivo, in vitro	mice	liver cells	inhibition of SYK to evaluate its role in inflammation	ALD, ALI, steatosis	EtOH ↑ levels of SYK in HCs and mononuclear cells, inhibition of SYK ↓ levels of neutrophil infiltration, immune cell activation and kinase 1- and kinase 2-mediated NF-κB activity	[229]
